# Effectiveness of nape acupuncture for post-stroke dysphagia: a meta-analysis and trial sequential analysis of randomized controlled trials

**DOI:** 10.3389/fneur.2026.1720302

**Published:** 2026-02-18

**Authors:** Hangyu Shi, Yonggang Yang, Huijuan Lou, Shaotao Chen

**Affiliations:** 1 Changchun University of Chinese Medicine, Changchun, China; 2 The First Affiliated Hospital of Changchun University of Chinese Medicine, Changchun, China

**Keywords:** acupuncture, dysphagia, meta-analysis, nape acupuncture, stroke, trialsequential analysis

## Abstract

**Background:**

This systematic review aimed to evaluate the efficacy and safety of nape acupuncture in improving swallowing function and quality of life in patients with post-stroke dysphagia (PSD), thereby providing evidence-based support for clinical treatment Strategies.

**Methods:**

A comprehensive literature search was conducted across eight databases: CNKI, VIP, WanFang, CBM, PubMed, Web of Science, Cochrane Library, and Embase. All randomized controlled trials (RCTs) investigating nape acupuncture for PSD, published from database inception to September, 2025, were included. Methodological quality was assessed using the Cochrane Risk of Bias tool for RCTs. Statistical analyses were performed using RevMan 5.4, Stata 17.0, and TSA software, including subgroup analyses, sensitivity analyses, and trial sequential analysis to identify sources of heterogeneity and assess the robustness of results. Adverse events were collected to provide data for evaluating the safety of nape acupuncture treatment, and the GRADE approach was used to assess the quality of the results.

**Results:**

A total of 21 eligible RCTs involving 1,995 participants were included in the meta-analysis. Results demonstrated that both nape acupuncture alone and nape acupuncture combined with other therapies significantly improved swallowing function in video fluoroscopic swallow study (VFSS): [Mean difference (MD) = 1.22, 95% confidence interval (CI) (0.94, 1.51), *p* < 0.00001], standardized swallowing assessment (SSA): [MD = −3.59, 95% CI (−4.35, −2.84), *p* < 0.00001], and total effective rate: [Odds ratio (OR) = 3.69, 95% CI (2.70, 5.04), *p* < 0.00001]. Moreover, it also exerted a positive effect on improving patients’ quality of life in swallowing quality of life questionnaire (SWAL-QOL): [MD = 13.42, 95% CI (9.46, 17.37), *p* < 0.00001], and barthel index (BI): [MD = 9.2, 95% CI (3.99, 14.4), *p* < 0.005]. TSA provides sufficient information to support the conclusion, but due to issues such as high heterogeneity, the GRADE system rates the quality of evidence as moderate to very low.

**Conclusion:**

Nape acupuncture, either alone or in combination with other therapies, significantly improves swallowing function and quality of life in patients with PSD, with a favorable safety profile characterized by minimal adverse events.

**Systematic review registration:**

https://www.crd.york.ac.uk/PROSPERO, identifier CRD420251089406.

## 1 Introduction

Stroke ranks as the second leading cause of death and one of the primary causes of disability worldwide, with over 11.9 million new cases occurring annually (1, 2). Post-stroke dysphagia (PSD) is one of the most common complications following stroke, affecting over 50% of stroke survivors (3). Dysphagia occurs when cerebrovascular lesions damage the muscles and neural structures, including the cerebral cortex, brainstem, and cranial nerves, involved in swallowing. This impairs the safe and efficient transport of food from the oropharynx to the stomach (4, 5). Clinical manifestations primarily include difficulty eating, choking on liquids, and speech disorders. It frequently occurs during the acute phase of stroke, leading to reduced quality of life and a series of severe consequences, including aspiration pneumonia, malnutrition, gastroesophageal reflux disease (GERD), anxiety, and depression, while also causing substantial economic losses (6). Multiple meta-analyses have found that acute stroke patients with PSD had a roughly 4.07-fold increased likelihood of dying and a significantly higher incidence of poor outcomes (7–9).

Current PSD therapies include swallowing training, neuromuscular electrical stimulation (NMES), transcranial direct current stimulation (tDCS), repetitive transcranial magnetic stimulation (rTMS), oral sensory stimulation, balloon dilation, behavioral motor therapy, and postural compensation (10). Even while these techniques are frequently employed in clinical settings and demonstrate some efficacy, they remain significantly limited. For instance, swallowing training requires a high level of patient compliance; some individuals struggle to persist due to cognitive impairments, fatigue, or poor cooperation, which undermines its actual efficacy (11). Muscle electrical stimulation is costly, and isolated muscle stimulation has limited effects on neural plasticity and central nervous system activation, which hinders primary disease treatment and compromises long-term rehabilitation outcomes (12, 13). Consequently, exploring a comprehensive intervention with sustained efficacy has become an urgent clinical need.

Acupuncture serves as a pillar of the theoretical framework of traditional Chinese medicine and embodies its philosophical principles. Given its advantages of affordability and favorable targeted therapeutic effects, acupuncture has become an effective complementary intervention in conventional clinical practice. Multiple international organizations and institutions have recognized its role in improving PSD symptoms (3, 14). However, there is currently no unified standard for the specific selection of acupoints and acupuncture prescriptions. Establishing precise acupoint prescriptions is necessary in order to address the standardization issues associated with different intervention protocols. In the academic field, the acupuncture technique targeting specific cervical regions and acupoints is collectively referred to as “nape acupuncture.” This term encompasses the selection of acupuncture points primarily located in the cervical region, including all relevant acupoints on both the anterior and posterior aspects of the neck, selected according to the pathogenesis of the disease. It is a distinctive acupuncture method primarily used for treating conditions involving the cervical region and adjacent intracranial structures (15). Due to the unique anatomical location of the neck acupoints, their positioning falls within the sensory fiber distribution areas of the glossopharyngeal and vagus nerves. It houses the brainstem, nerves, vertebral arteries, and muscle groups involved in swallowing, serving as a vital pathway that connects the central nervous system to peripheral nerves and muscles (16). Considering this anatomical characteristic, some research suggests that nape acupuncture can also trigger neural impulses to the central nervous system, which may aid in brain tissue neuronal repair, rebuild the swallowing reflex arc, and enhance neural repair and synaptic plasticity, thereby achieving the goal of alleviating PSD symptoms (17).

In recent years, two meta-analyses on similar topics have been published. Although both confirmed the positive effect of nape acupuncture in treating PSD, the strength of evidence remains limited. Tang et al.’s study (18) only compared cervical acupuncture with swallowing training and did not address whether nape acupuncture plays a positive role when combined with other interventions. Furthermore, multiple studies reported identical outcome values, raising concerns about the inclusion of duplicate literature. Hu et al.’s study (19) focused solely on efficacy rates and cure rates as outcome measures, raising concerns about excessive subjectivity. Therefore, this study included both studies using nape acupuncture alone and those combining it with other interventions. Although distal acupoints such as those located on the lower extremities are clinically employed in the treatment of this condition, to ensure the specificity of the research topic and control the homogeneity of the intervention, studies involving other acupoints were excluded, with only those focusing on nuchal acupoint interventions being included in the present analysis. While incorporating multiple scales and the gold standard VFSS as outcome measures. Additionally, meta-analyses inherently increase the risk of Type I errors (false positives) due to systematic or random errors, which can potentially lead to an overestimation of treatment efficacy. Trial sequence analysis (TSA) was conducted to control for the risk of false positives and the required information size (RIS) in the cumulative evidence, thereby enhancing the reliability of the findings (20). This study aims to accurately evaluate the clinical efficacy of nape acupuncture for PSD and its impact on quality of life, providing evidence-based medical support for clinical research.

## 2 Methods

### 2.1 Protocol and registration

This systematic review protocol was developed in accordance with the Preferred Reporting Items for Systematic Reviews and Meta-Analyses (PRISMA) guidelines (21). It has been registered in PROSPERO with the registration number is CRD420251089406.

### 2.2 Search strategy

The search date range spans from the establishment of each database until August 23, 2025. We searched eight databases, including four Chinese databases and four English databases. The databases are as follows: China National Knowledge Infrastructure (CNKI), Chinese Science and Technology Journal (VIP), WanFang, China Biology Medicine (CBM), PubMed, Web of Science, Cochrane Library, and Embase. No language or country restrictions were applied. Chinese subject headings and free-text terms were determined using subject headings from the SinoMed database. English subject headings and free-text terms were determined using the MeSH database from the PubMed database. Search terms included “stroke,” “Deglutition Disorders,” “nape acupuncture,” and “randomized controlled trial.” The specific search strategies are in the Supplementary materials.

### 2.3 Inclusion criteria

The following are the inclusion criteria that were developed using the PICOS principles:

(1) Participants: Patients diagnosed with PSD, with imaging findings consistent with PSD diagnosis. No restrictions were imposed on stroke type, age, or disease duration. (2) Interventions: The treatment group received nape acupuncture alone or in combination with the control group’s treatment. In terms of experimental design, the only difference between the two groups should be the application of nape acupuncture. No restrictions were imposed on treatment duration, total number of sessions, or selected acupoints; (3) Control Groups: Interventions such as conventional acupuncture, swallowing exercises, or electrical muscle stimulation; (4) Outcome Measures: Include the video fluoroscopic swallow study (VFSS), total effective rate. Swallowing Quality of Life Questionnaire (SWAL-QOL), and Barthel Index (BI); (5) Study Design: Only randomized controlled trials (RCTs) were included.

### 2.4 Exclusion criteria

Tables should be inserted at the end of the manuscript. (1) Studies with duplicate publications or overlapping data; (2) Ineligible study types, including reviews, meta-analyses, conference abstracts, case reports, and animal experiments; (3) The experimental design, including intervention measures or grouping methods, does not meet the inclusion criteria; (4) Studies with unclear diagnostic or therapeutic efficacy assessment criteria; (5) Studies that lack full-text access or whose information is invalid or incomplete.

### 2.5 Data extraction

After completing the search, the retrieved literature was imported into EndNote 21.0 for unified screening and management according to predefined inclusion and exclusion criteria. Two researchers (LHJ, SHY) conducted independent, blinded literature screening. They first performed an initial screening by reviewing article titles and abstracts, then downloaded full texts for a secondary screening. The original authors were contacted whenever possible to provide further information when data were lacking. In cases of unresolved disagreement, the issue was referred to a third researcher (YYG) for adjudication. Extracted information included: (1) Basic information: publication date, authors, randomization method, and blinding status; (2) Patient information: sample size, patient age, and disease duration; (3) Intervention details: treatment modality, acupoint prescription, number of sessions, and treatment duration; (4) Outcome measures and adverse events.

### 2.6 Quality assessment

The Cochrane Risk of Bias 2.0 (RoB2.0) tool was used to systematically assess potential bias in the included studies (22). This tool evaluates each study across multiple domains, specifically: randomisation procedures, intervention implementation, missing outcome data, outcome measurement, selective reporting, and overall risk of bias. For each domain, risk of bias was assessed as “low risk,” “high risk,” or “some concerns” based on criteria outlined in the tool manual. Two researchers conducted independent assessments; disagreements were resolved through discussion or consultation with a third researcher. Risk of bias assessments were visualized using risk of bias plots and summary plots.

### 2.7 Statistical analysis

Statistical analysis of the included data was performed using RevMan 5.4 and Stata 17.0 software. For continuous variables, the mean difference (MD) was used as the pooled effect measure, with outcomes assessed using 95% confidence intervals (95% CI). For dichotomous variables, the odds ratio (OR) was used as the pooled effect measure, with outcomes assessed using 95% CI. Heterogeneity was assessed using the *Q* test and *I*^2^ statistic. If I^2^ ≤ 50% and *p* ≥ 0.1, indicating low heterogeneity, a fixed-effect model was used to combine effect sizes. If I^2^ > 50% or *p* < 0.1, indicating substantial heterogeneity, a random-effects model was used for pooling. Subgroup analyses were then conducted based on treatment duration and control group intervention type to explore sources of heterogeneity. Sensitivity analyses were performed by sequentially excluding studies to assess the robustness of results. For outcomes with numerous included studies (n ≥ 10), Egger’s test and funnel plots were used to assess potential publication bias.

### 2.8 Trial sequential analysis

TSA 0.9.5.10 Beta software^1^ was used for a sequential analysis of VFSS scores and the total effective rate. In this study, a 5% alpha error and a 20% beta error were assumed, with the application of a random-effects model. This analysis avoided the risk of false positives (Type I errors) arising from sparse data and repeated testing of cumulative data by employing pre-specified conventional boundaries and trial sequential monitoring boundaries (TSMBs). Simultaneously, it assessed whether the current cumulative evidence was sufficient by calculating the required information size (RIS) and evaluated the validity of the evidence through the construction of cumulative Z-curves. If the Z-curve crosses both the conventional boundary and the TSMB boundary, it indicates robust conclusions and validated validity. Failure to reach the RIS suggests insufficient current evidence, necessitating further data support.

### 2.9 Results evaluation

We used GRADEpro GDT software (GRADEpro GDT 2023) to assess evidence quality across five GRADE domains: risk of bias, inconsistency, indirectness, imprecision, and publication bias. Evidence quality was downgraded when limitations were identified (22).

## 3 Results

Using predefined search and screening criteria, we retrieved 509 articles from eight databases, both in Chinese and English. Based on title and abstract information, 476 articles were excluded due to duplication or failure to meet inclusion criteria. After downloading and reviewing full texts, an additional 12 articles were excluded for non-compliance with the inclusion criteria. Ultimately, 21 articles were included in the analysis.

### 3.1 Screening process and results

**Figure 1:**
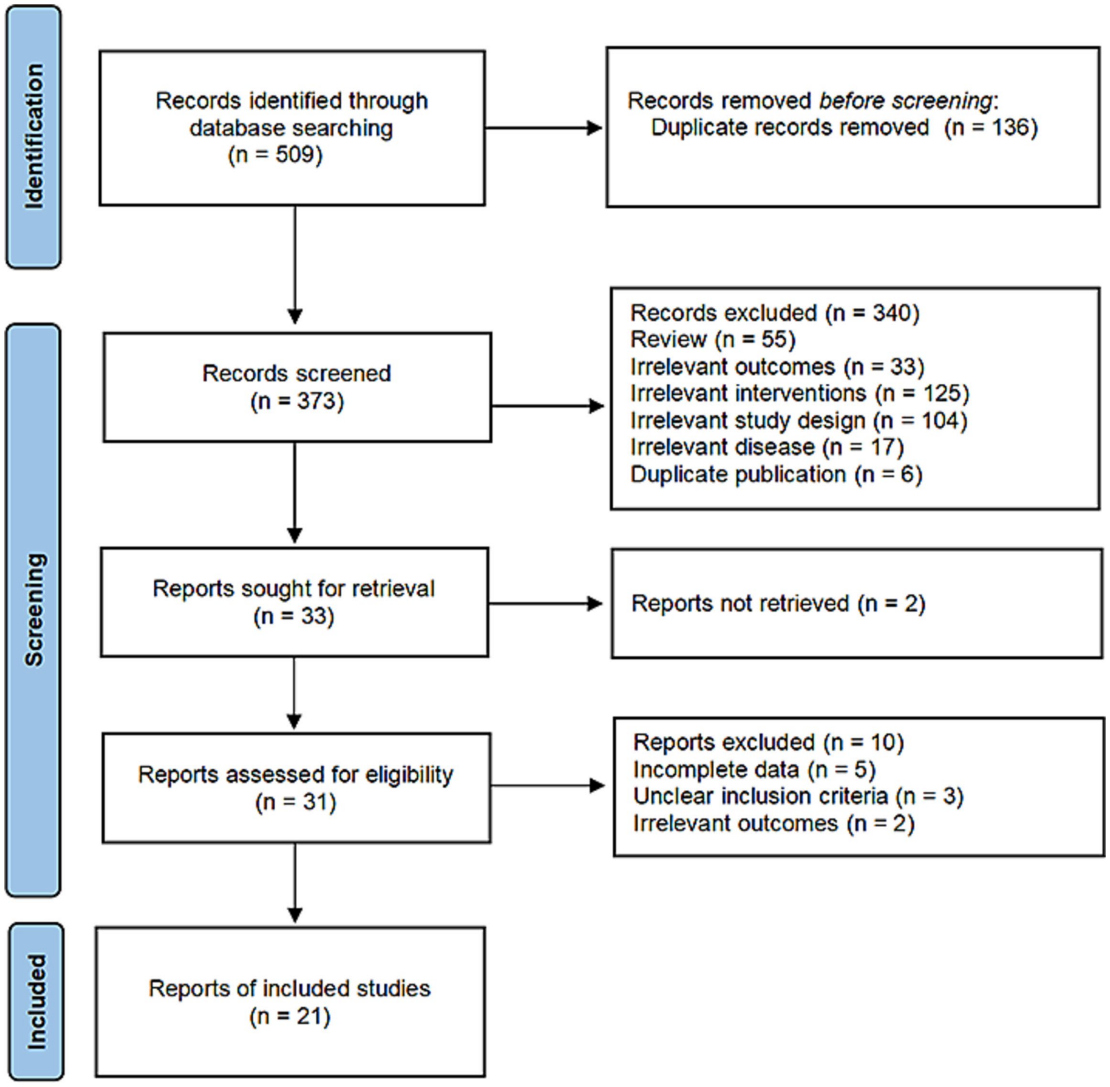
Literature screening flowchart.

Using predefined search and screening criteria, we retrieved 509 articles from eight databases, both in Chinese and English. Based on title and abstract information, 476 articles were excluded due to duplication or failure to meet inclusion criteria. After downloading and reviewing full texts, an additional 12 articles were excluded for non-compliance with the inclusion criteria. Ultimately, 21 articles were included in the analysis. The screening process is detailed in Figure 1.

### 3.2 Study characteristics

A total of 21 studies were included in the analysis (23–43). All studies were conducted by Chinese research teams, involving a total of 1,995 patients. The studies spanned the period from 2009 to 2025. All were two-arm trials with comparable baseline data between the treatment and control groups, ensuring comparability between groups. Among these, 18 studies employed nape acupuncture as the intervention in the treatment group (23–31, 33, 34, 37–43), while 3 studies used electric nape acupuncture (32, 35, 36). Eleven studies used swallowing training as the control intervention (23, 25, 26, 29–31, 33, 34, 41–43), two used NMES, two combined swallowing training with NMES (24, 35), one used transcranial magnetic stimulation (27), one used balloon dilation (32), and four used other acupuncture therapies (body acupuncture, intradermal acupuncture, tongue acupuncture) (28, 37–39). The characteristics of the study are detailed in Table 1.

**Table 1:** Literature basic feature extraction table.

Study	Age	Duration of disease	Sample size	Intervention	Acupoints	Treatment dosage	Outcome
Chu et al. (23)	T:67 ± 11	T:(41.1 ± 38.6)d	T:48	T: NA + ST	Fengchi (GB20), Yiming (EX-HN14), Gongxue (Extra),	Retained for 30 min	①④⑤
	C:67 ± 10	C:(40.5 ± 30.8)d	C:49	C: ST	Zhiqiang (Extra), Tunyan (Extra), Fayin (Extra),	5 times a week for 8 weeks	
					Lianquan (CV23), Waijinjin (Extra), Waiyuye (Extra)		
Gao and Zhou (24)	T:65 ± 5	T:(3.3 ± 1.5)m	T:30	T: NA + ST + NMES	Fengchi (GB20), Tianzhu (BL10), Wangu (GB12),	Retained for 30 min,	③④
	C:64 ± 5	C:(3.4 ± 1.6)m	C:30	C: ST + NMES	Lianquan (CV23), Pang Lianquan (Extra), Jinjin (EX-HN12),	5 times a week for 4 weeks	
					Yuye (EX-HN13)		
Guo and Li (25)	T:66.21 ± 8.03	T:(43.01 ± 5.33)d	T:50	T: NA + ST	Yamen (DU15), Tianzhu (BL10), Zhiqiang (Extra),	Retained for 30 min,	②④⑤
	C:65.91 ± 7.85	C:(43.45 ± 5.27)d	C:50	C: ST	Tianzhu (BL10), Fengfu (DU16), Lianquan (CV23)	for 4 weeks	
He et al. (26)	T:64 ± 6	T:(32 ± 15)d	T:34	T: ENA + ST	Cervical Huatuo Jiaji Points (C2-C), Fengchi (GB20),	Retained for 30 min,	⑤
	C:69 ± 7	C:(27 ± 15)d	C:35	C: ST	Lianquan (CV23)	5 times a week for 6 weeks	
Jiang et al. (27)	T:51.23 ± 6.34	T:(7.04 ± 1.56)d	T:60	T: NA + TMS	Renying (ST9), Lianquan (CV23), Fengchi (GB20),	6 times a week for 3 weeks	④⑤
	C:50.97 ± 6.41	C:(7.21 ± 0.91)d	C:60	C: TMS	Gongxue (Extra), Yifeng (TE17), Fengfu (DU16)		
Li et al. (28)	T:62.63 ± 9.37	T:(62.70 ± 72.58)d	T:30	T: NA	BA, Yamen (DU15), Tianzhu (BL10), Zhiqiang (Extra)	Retained for 30 min,	⑤
	C:63.61 ± 8.69	C:(62.03 ± 71.79)d	C:30	C: BA		6 times a week for 4 weeks	
Li et al. (29)	T:61.9 ± 7.9	T:(16.9 ± 7.1)d	T:40	T: NA + ST	Fengchi (GB20), Yiming (EX-HN14), Gongxue (Extra),	Retained for 30 min,	④⑤
	C:63. ± 6.9	C:(18.5 ± 8.1)d	C:40	C: ST	Zhiqiang (Extra), Lianquan (CV23), Waijinjin (Extra),	6 times a week for 4 weeks	
					Waiyuye (Extra)		
Lin et al. (30)	T:68.43 ± 6.12	T:(15.61 ± 3.74)d	T:88	T: NA + ST	Fengfu (DU16), Fengchi (GB20), Wangu (GB12),	6 times a week for 3 weeks	①④
	C:68.21 ± 6.34	C:(15.37 ± 3.85)d	C:88	C: ST	Yifeng (TE17), Buxue (Extra), Jingbailao (EX-HN15)		
Liu et al. (31)	T:66.2 ± 11.3	T:(42 ± 1.17)d	T:47	T: NA + ST	Fengchi (GB20), Yiming (EX-HN14), Gongxue (Extra),	Retained for 30 min,	①④⑤
	C:65.9 ± 10.9	C:(32 ± 3.16)d	C:47	C: ST	Zhiqiang (Extra), Tunyan (Extra), Fayin (Extra),	5 times a week for 8 weeks	
					Lianquan (CV23), Waijinjin (Extra), Waiyuye (Extra)		
Long et al. (32)	T:60.53 ± 10.61	T:(21.73 ± 18.07)d	T:30	T: ENA + CBD	Lianquan (CV23), Pang Lianquan (Extra), Yifeng (TE17),	Retained for 30 min,	④⑤
	C:59.93 ± 12.89	C:(22.07 ± 16.74)d	C:30	C: CBD	Fengchi (GB20)	6 times a week for 4 weeks	
Qi et al. (33)	T:63 ± 10	T:(14.2 ± 4.1)d	T:60	T: NA + ST	Fengchi (GB20), Tianzhu (BL10), Wangu (GB12),	Retained for 30 min,	③④⑤
	C:63 ± 11	C:(15.2 ± 3.8)d	C:60	C: ST	Lianquan (CV23), Pang Lianquan (Extra), Jinjin (EX-HN12),	7 times a week for 2 weeks	
					Yuye (EX-HN13)		
Qin et al. (34)	T:47.1 ± 6.3	T:(45.2 ± 7.8)d	T:50	T: NA + ST	Lianquan (CV23), Gongxue (Extra), Renying (ST9)	Retained For 30 min,	③⑤
	C:46.1 ± 6.9	C:(44.9 ± 8.6)d	C:50	C: ST		for 20 days	
Song et al. (35)	T:67.27 ± 4.51	T:(17.5 ± 3.87)m	T:45	T: ENA + ST + NMES	Cervical Huatuo Jiaji Points (C2-C6)	Retained For 30 min,	④
	C:67.35 ± 4.72	C:(17.79 ± 4.26)m	C:45	C: ST + NMES		3 days on, 1 day off for 2 months	
Wang et al. (36)	T:66.21 ± 12.54	T:(33.99 ± 2.42)d	T:72	T: ENA + ST	Fengfu (DU16), Fengchi (GB20), Tianzhu (BL10),	Retained For 30 min,	②
	C:65.45 ± 12.27	C:(34.44 ± 2.51)d	C:88	C: ST	Yiming (EX-HN14), Gongxue (Extra), Tunyan (Extra),	6 times a week for 8 weeks	
					Zhiqiang (Extra), Lianquan (CV23), Lianquan (CV23)		
Wang et al. (37)	T:61.05 ± 4.61	T:(5.47 ± 1.01)m	T:40	T: NA + IN	IN, Wangu (GB12), Tianzhu (BL10), Lianquan (CV23),	Retained for 30 min,	④⑤
	C:60.28 ± 4.19	C:(5.63 ± 1.22)m	C:40	C: IN	Fengchi (GB20), Pang Lianquan (Extra), Jinjin (EX-HN12),	for 2 weeks	
					Yuye (EX-HN13)		
Wang et al. (38)	T:62.86 ± 7.26	T:(5.72 ± 1.68)m	T:30	T: NA + BA	BA, Yamen (DU15), Fengfu (DU16), Xianaohu (Extra),	Retained for 30 min,	②⑤
	C:63.49 ± 5.74	C:(5.31 ± 1.46)m	C:30	C: BA	Pang Lianquan (Extra)	6 times a week for 21 days	
Wei et al. (39)	T:51.41 ± 6.16	T:(17.45 ± 3.73)d	T:49	T: NA + TA	TA, Tianzhu (BL10), Fengchi (GB20), Wangu (GB12),	Retained for 30 min,	①③⑤
	C:51.53 ± 6.22	C:(17.53 ± 3.77)d	C:49	C: TA	Pang Lianquan (Extra), Lianquan (CV23), Jinjin (EX-HN12),	5 times a week for 4 weeks	
					Yuye (EX-HN13)		
Yang et al. (40)	T:62.44 ± 7.92	T:(42.98 ± 4.82)d	T:44	T: NA + NMES	Yamen (DU15), Fengfu (DU16), Tianzhu (BL10),	Retained for 30 min,	②④⑤
	C:62.05 ± 7.88	C:(43.01 ± 5.56)d	C:44	C: NMES	Lianquan (CV23), Zhiqiang (Extra)	5 times a week for 4 weeks	
Zhang et al. (41)	T:67.03 ± 6.25	T:(64.12 ± 9.26)d	T:62	T: NA + ST	Yamen (DU15), Tianzhu (BL10), Zhiqiang (Extra),	Retained for 30 min,	③④⑤
	C:66.81 ± 6.03	C:(63.94 ± 8.93)d	C:61	C: ST	Fengfu (DU16), Lianquan (CV23)	5 times a week for 4 weeks	
Zhao et al. (42)	T:64.18 ± 2.12	T:(15.27 ± 2.65)d	T:40	T: NA + NMES	Fengchi (GB20), Gongxue (Extra), Tunyan (Extra),	Retained for 20 min,	④⑤
	C:64.02 ± 2.27	C:(15.15 ± 2.77)d	C:40	C: NMES	Yifeng (TE17), Fengfu (DU16), Lianquan (CV23),	6 times a week for 4 weeks	
					Jinjin (EX-HN12), Yuye (EX-HN13)		
Zhou et al. (43)	T:57 ± 8	T:(10.5 ± 3.8)d	T:40	T: NA + ST	Fengchi (GB20), Yiming (EX-HN14), Gongxue (Extra),	Retained for 30 min,	③④⑤
	C:58 ± 7	C:(15.1 ± 7.7)d	C:40	C: ST	Zhiqiang (Extra), Tunyan (Extra), Lianquan (CV23),	6 times a week for 4 weeks	
					Waijinjin (Extra), Waiyuye (Extra)		

T, treatment; C, Control; NA, Nape Acupuncture; ENA, Electric Nape Acupuncture; BA, Body Acupuncture; TA, Tongue Acupuncture; IN, IntradermalNeedle; ST, Swallowing Training; NMES, Neuromuscular Electrical Stimulation; CBD,catheter balloon dilatation; ① Swallowing-Quality of Life (SWAL-QOL); ② Barthel Index (BI); ③ video fluoroscopic swallowing study (VFSS); ④ standardized swallowing assessment (SSA); ⑤ Total effective rate.

### 3.3 Risk of bias in studies

**Figure 2:**
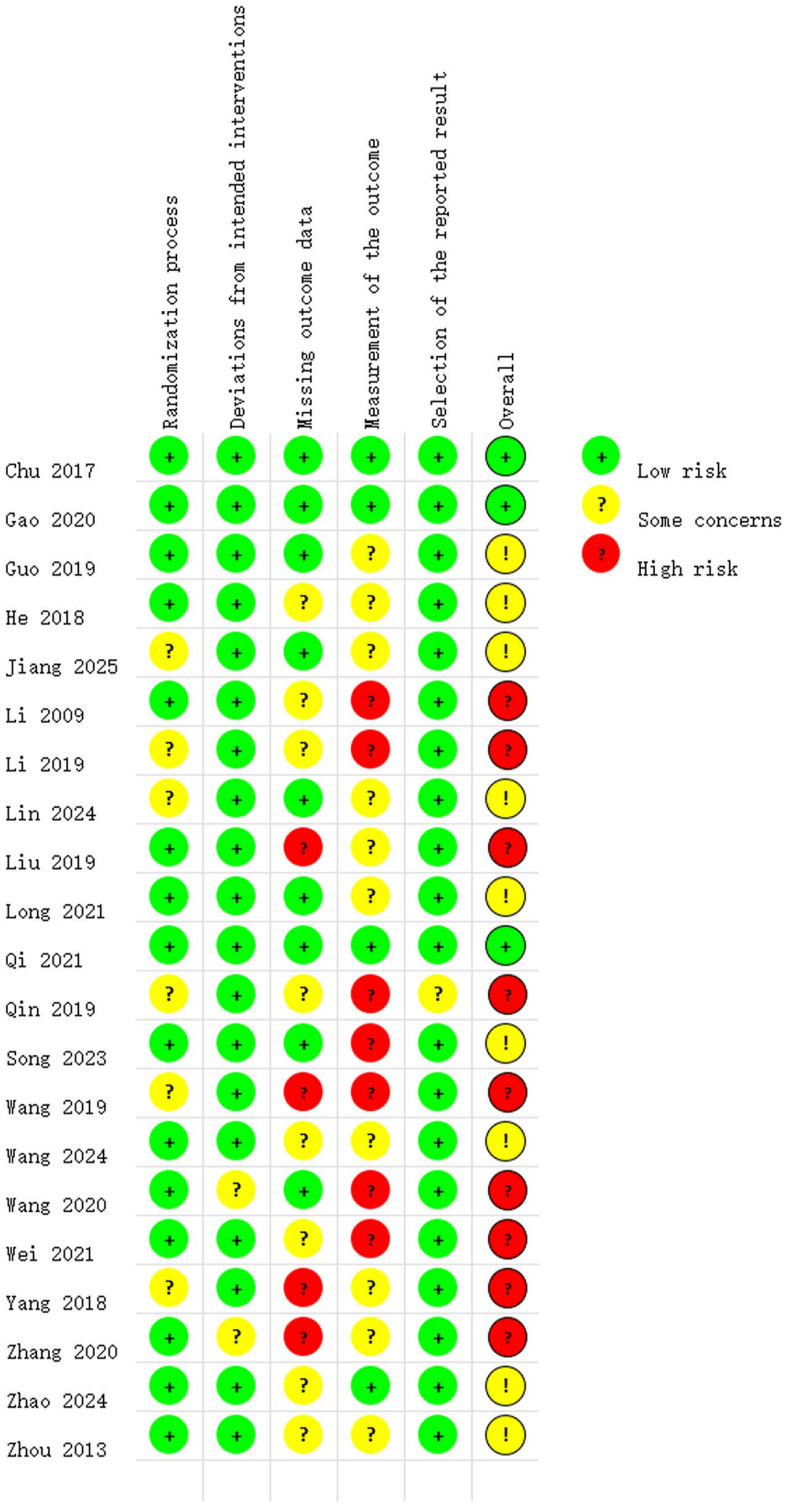
Risk of bias summary.

**Figure 3:**
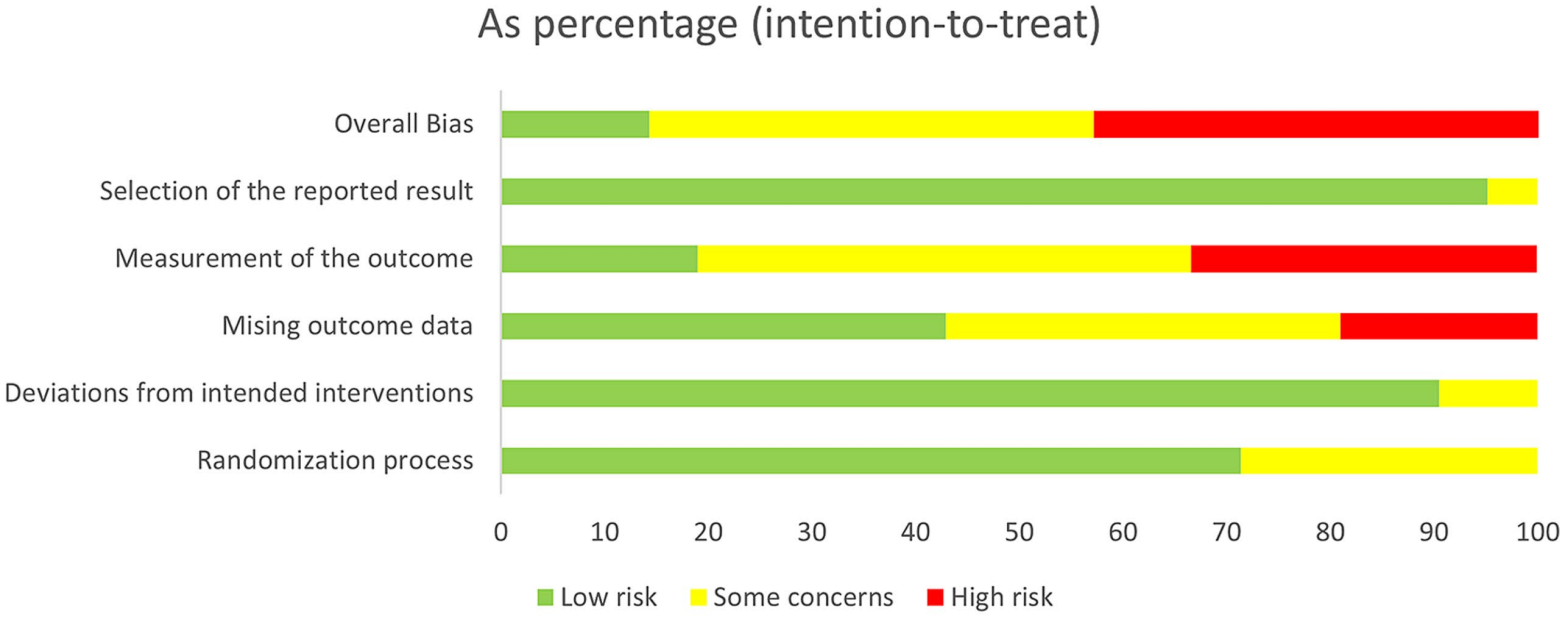
Risk of bias graph.

Following the assessment of bias risk using RoB2.0, it was found that 13 studies exhibited moderate or low risk of bias, while 9 studies demonstrated a higher risk of bias. The variation in risk of bias is primarily related to outcome data attrition or outcome measurement. See Figures 2, 3 for details.

### 3.4 Meta-analysis results

#### 3.4.1 Video fluoroscopic swallow study

**Figure 4:**
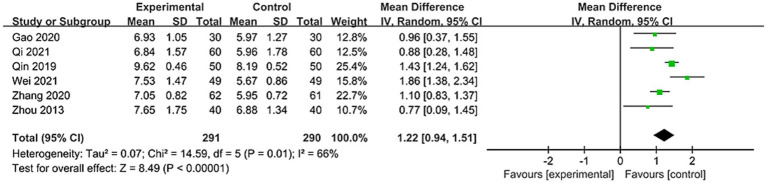
Forest plot of VFSS comparison between two groups.

Among the 21 studies, 6 studies reported VFSS scores involving 581 patients (24, 33, 34, 39, 41, 43). As shown in Figure 4, due to significant heterogeneity between studies (*I*^2^ = 66%), a random-effects model was applied. Results demonstrated a statistically significant difference between the two groups [MD = 1.22, 95% CI (0.94, 1.51), *p* < 0.00001]. This indicates that using nape acupuncture alone or as part of a combination therapy offers a greater advantage in improving VFSS scores compared to the control group.

**Figure 5:**
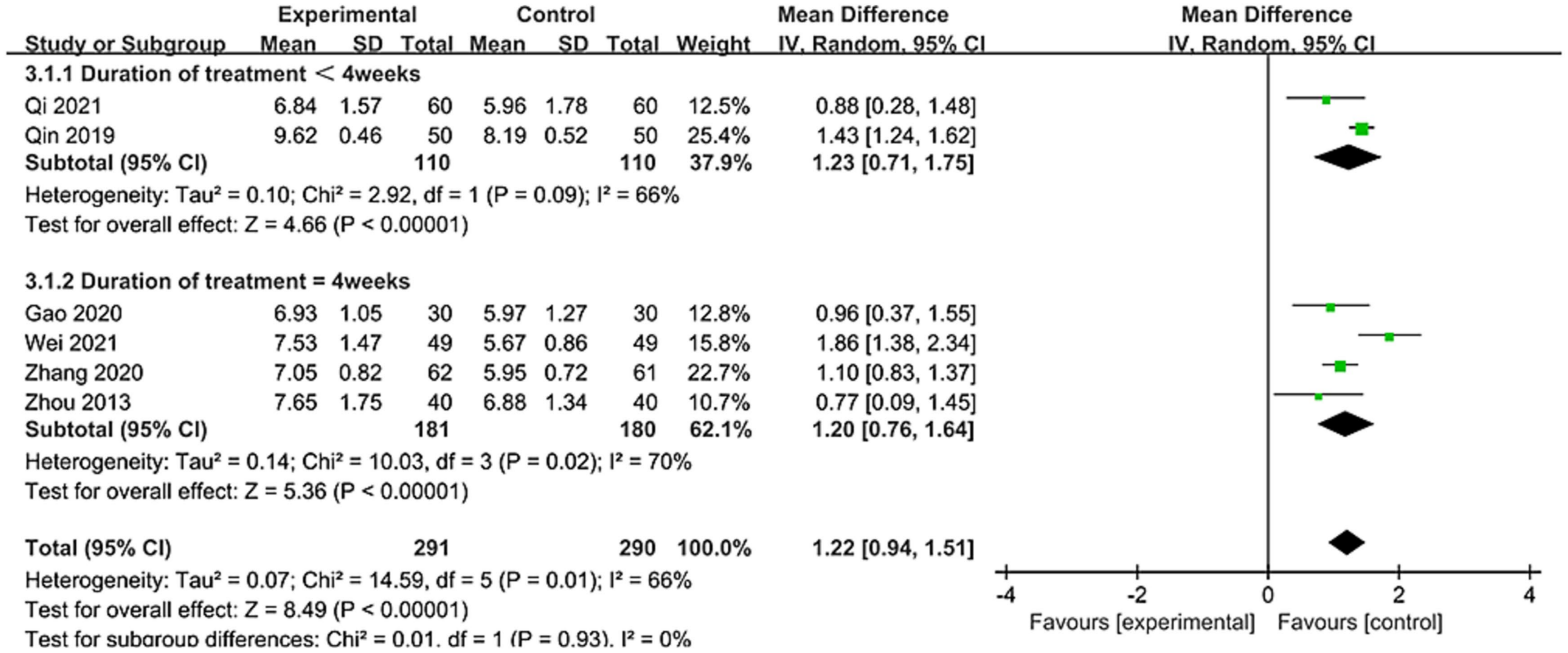
Forest plot of VFSS subgroup stratified by treatment duration subgroup.

To identify sources of heterogeneity, subgroup analyses were conducted based on treatment duration and control group interventions. Treatment duration subgroups were categorized as short-term (<4 weeks) and long-term (=4 weeks). Results showed no significant differences between subgroups (*p* = 0.93, *I*^2^ = 0%). For subgroup with treatment duration <4 weeks: [MD = 1.23, 95% CI (0.71, 1.75), *p* < 0.00001]; For subgroup with treatment duration = 4 weeks: [MD = 1.20, 95% CI (0.76, 1.64), p < 0.00001], see Figure 5 for details. This indicates that both treatment subgroups outperformed the control group in improving VFSS scores, demonstrating superior enhancement of swallowing function.

**Figure 6:**
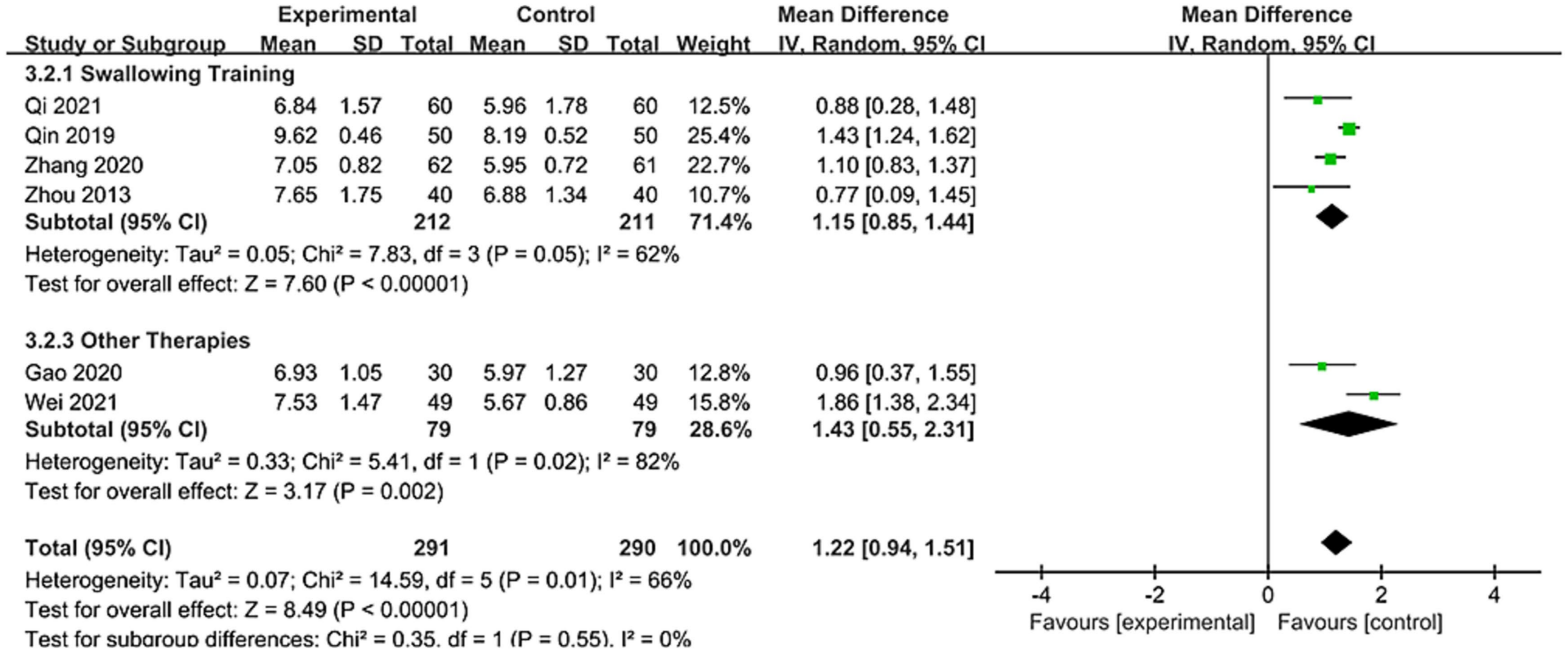
Forest plot of VFSS subgroup by control group interventions.

The control group intervention subgroups were divided into swallowing training and other therapies (swallowing combined with electrical stimulation, tongue acupuncture). Results showed no significant differences between subgroups (*p* = 0.55, *I*^2^ = 0%). The swallowing training subgroup demonstrated [MD = 1.15, 95% CI (0.85, 1.44), *p* < 0.00001], while the other therapies subgroup showed [MD = 1.43, 95% CI (0.55, 2.31), *p* < 0.05], see Figure 6 for details. This indicates that both subgroups outperformed the control group in improving VFSS scores. To ensure robustness, sensitivity analyses were conducted (see Supplementary Figures). No single study had a significant influence on the results or heterogeneity. Thus, treatment duration or control group interventions were not considered primary sources of heterogeneity. When the studies by Qin et al. (34) and Wei (39), which included relatively younger patients, were analyzed separately as a subgroup, heterogeneity dropped to 0%. This suggests age differences among patients across studies may be a potential source of heterogeneity.

#### 3.4.2 Standardized swallowing assessment

**Figure 7:**
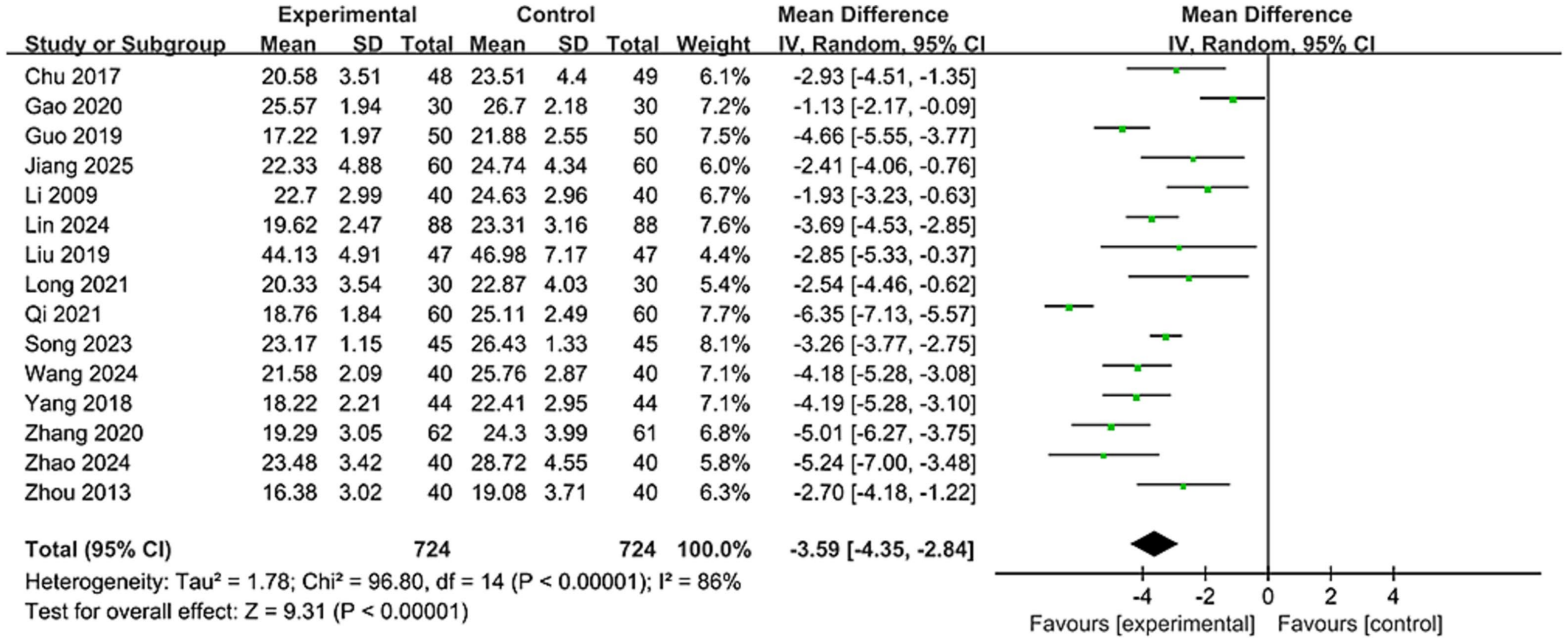
Forest plot of SSA comparison between two groups.

A total of 15 studies reported SSA scores, involving 1,448 patients (23–25, 27, 28, 30–33, 35, 37, 40–43). As shown in Figure 7, due to significant heterogeneity among studies (*I*^2^ = 86%), a random-effects model was applied. Results demonstrated a statistically significant difference between groups [MD = −3.59, 95% CI (−4.35, −2.84), *p* < 0.00001]. This indicates that using nape acupuncture alone or as part of a combination therapy offers a greater advantage in reducing SSA scores compared to the control group.

Subgroups were categorized by treatment duration: ≤4 weeks and >4 weeks. Results showed no significant difference between subgroups (*p* = 0.37, *I*^2^ = 0%). For subgroup with treatment duration>4 weeks: [MD = -3.21, 95% CI (−3.69, −2.74), p < 0.00001]; and for subgroup with treatment duration≤4 weeks: [MD = −3.71, 95% CI (−4.66, −2.76), p < 0.00001], see Supplementary Figures. Indicating both subgroups outperformed the control group in reducing SSA scores. Furthermore, heterogeneity disappeared in the subgroup with treatment duration>4 weeks (*p* = 0.89, *I*^2^ = 0%), suggesting that heterogeneity may be related to treatment duration.

The control group intervention subgroups included swallowing training, NMES, and other therapies (combined swallowing and electrical stimulation, transcranial electrical stimulation, balloon dilation, intradermal needles). Results showed significant differences between subgroups (*p* = 0.05, *I*^2^ = 65.9%). The swallowing training subgroup showed [MD = -3.87, 95% CI (−4.99, −2.75), *p* < 0.00001]; the electrical stimulation subgroup showed [MD = −4.48, 95% CI (−5.41, −3.55), *p* < 0.00001], and the other therapies subgroup showed [MD = −2.75, 95% CI (−3.81, −1.69), *p* < 0.00001], see Supplementary Figures. This indicates that both subgroups outperformed the control group in improving VFSS scores, demonstrating superior enhancement of swallowing function. Furthermore, heterogeneity disappeared in the electrical stimulation group (*p* = 0.32, *I*^2^ = 0%), suggesting that differing intervention approaches within the control group, as well as the combination of treatment groups with various intervention modalities, contributed to the observed heterogeneity. To ensure the robustness of the results, a sensitivity analysis (see Supplementary Figures) revealed that no single study significantly affected the outcomes or heterogeneity.

#### 3.4.3 Swallowing quality of life questionnaire

**Figure 8:**
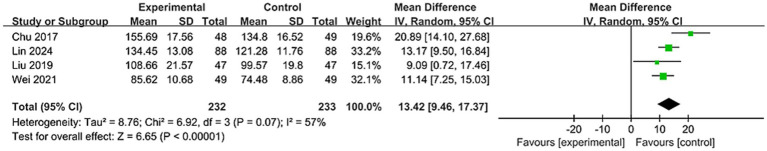
Forest plot of SWAL-QOL comparison between two groups.

A total of four studies reported SWAL-QOL scores involving 465 patients (23, 30, 31, 39). As shown in Figure 8, due to significant heterogeneity among studies (*I*^2^ = 57%), a random-effects model was applied. Results demonstrated a significant difference between groups [MD = 13.42, 95% CI (9.46, 17.37), *p* < 0.00001]. This indicates that using nape acupuncture alone or as part of a combination therapy offers a greater advantage in improving SWAL-QOL scores compared to the control group.

Subgroups were categorized by treatment duration: ≤4 weeks and >4 weeks. Results showed no significant difference between subgroups (*p* = 0.61, *I*^2^ = 0%). For the subgroup with treatment duration >4 weeks: [MD = 15.25, 95% CI (3.7, 26.81), *p* < 0.05]; for the subgroup with treatment duration ≤4 weeks: [MD = 12.21, 95% CI (9.54, 14.88), *p* < 0.00001], see Supplementary Figures. Indicating that both subgroups significantly improved SWAL-QOL scores compared to the control group, demonstrating superior quality of life enhancement. Heterogeneity disappeared in the subgroup with treatment duration ≤4 weeks (*p* = 0.46, *I*^2^ = 0%), suggesting treatment duration may influence heterogeneity.

The control group intervention subgroups were divided into swallowing training and other therapies (intradermal needles). Results showed no significant differences between subgroups (*p* = 0.34, *I*^2^ = 0%). The swallowing training subgroup had [MD = 14.52, 95% CI (8.69, 20.35), *p* < 0.00001]; while the other therapies subgroup showed [MD = 11.14, 95% CI (7.25, 15.03), *p* < 0.00001], see Supplementary Figures. Indicating both subgroups improved patients’ quality of life. To ensure robustness, a sensitivity analysis (see Supplementary Figures) revealed that no studies significantly affected the results or heterogeneity.

#### 3.4.4 Barthel index

**Figure 9:**
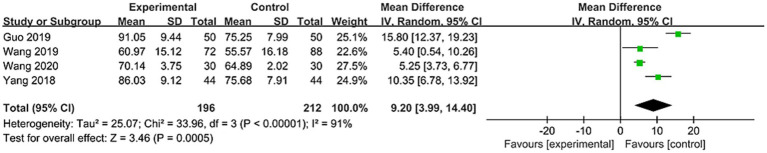
Forest plot of BI comparison between two groups.

A total of four studies reported BI scores involving 408 patients (25, 36, 38, 40). As shown in Figure 9, due to significant heterogeneity among studies (*I*^2^ = 97%), a random-effects model was applied. Results demonstrated a significant difference between groups [MD = 9.2, 95% CI (3.99, 14.4), *p* < 0.005]. This indicates that nape acupuncture alone or as part of combination therapy offers a greater advantage in improving BI scores compared to the control group.

Subgroups were categorized by treatment duration: ≤4 weeks and >4 weeks. Results showed no significant difference between subgroups (*p* = 0.24, *I*^2^ = 28.7%). For the subgroup with treatment duration >4 weeks: [MD = 10.34, 95% CI (3.77, 16.9), *p* < 0.00001]; for the subgroup with treatment duration ≤4 weeks: [MD = 5.4, 95% CI (0.54, 10.26), *p* < 0.03], see Supplementary Figures. This indicates that both subgroups outperformed the control group in improving BI scores, demonstrating superior enhancement of patients’ quality of life.

The control group intervention methods were subdivided into swallowing training and other therapies (body acupuncture, muscle electrical stimulation). Results showed no significant differences between subgroups (*p* = 0.58, *I*^2^ = 0%). The swallowing training subgroup showed [MD = 10.75, 95% CI (0.56, 20.94), *p* < 0.05], while the other therapies subgroup showed [MD = 7.53, 95% CI (2.56, 12.51), p < 0.05], see Supplementary Figures. This indicates that both subgroups can enhance patients’ quality of life. To ensure the robustness of the results, a sensitivity analysis was conducted (see Supplementary Figures). No single study had a significant influence on the results or heterogeneity. Thus, treatment duration or control group interventions were not considered primary sources of heterogeneity. When Guo and Li (25) and Yang et al.’s (40) study employing specific needle manipulation techniques was grouped separately, heterogeneity dropped to 0%. This suggests heterogeneity likely stems from variations in specific needle manipulation techniques within different acupuncture prescriptions.

#### 3.4.5 Total effective rate

**Figure 10:**
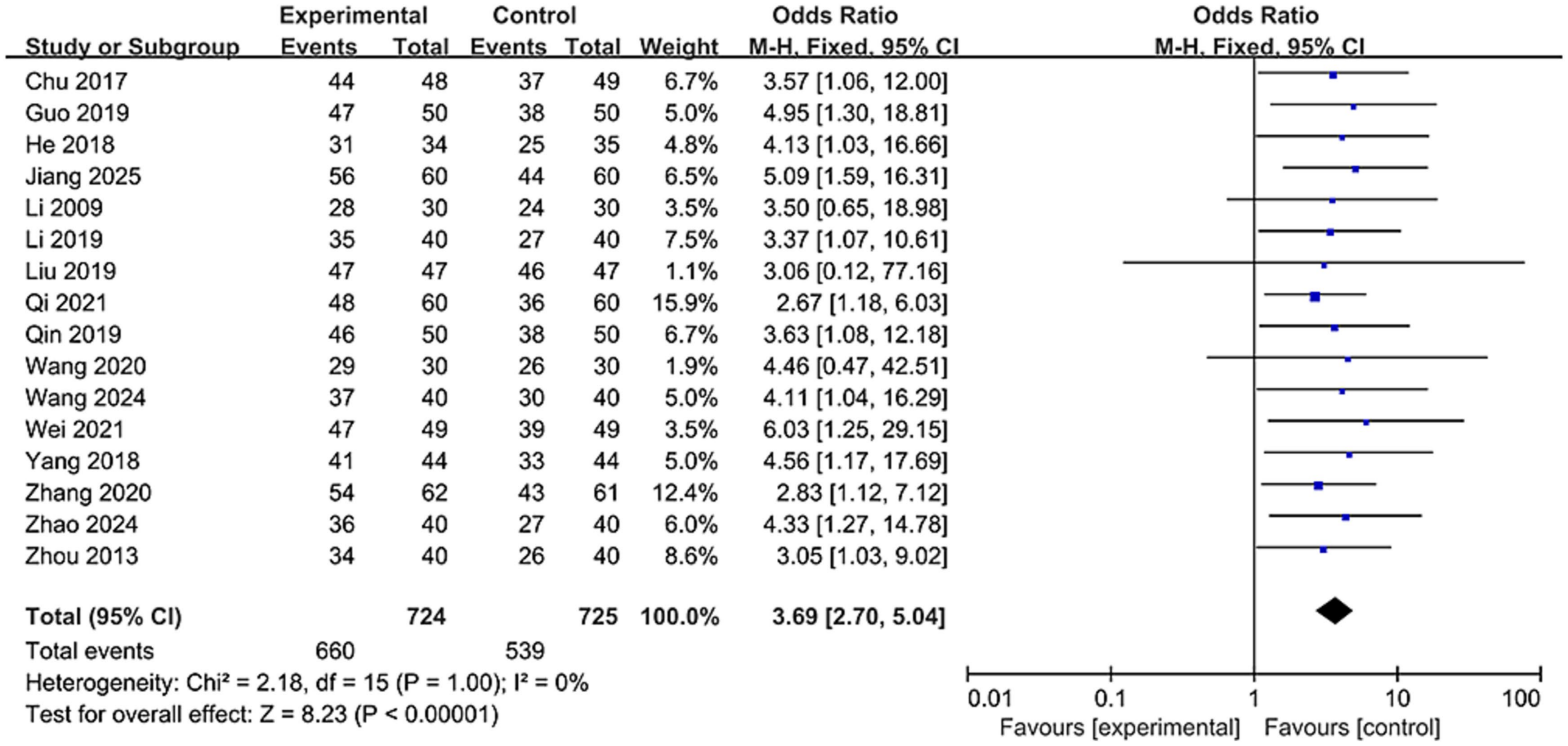
Forest plot of total effective rates comparison between two groups.

A total of 16 studies reported total effective rate, involving 1,449 patients (23, 25–29, 31, 33, 34, 37–43). The odds ratio (OR) was used as the pooled effect measure. As shown in Figure 10, due to negligible heterogeneity among studies (*I*^2^ = 0%), a fixed-effect model was applied. Results showed a significant difference between the two groups [OR = 3.69, 95% CI (2.7, 5.04), *p* < 0.00001]. This suggests that nape acupuncture, as a treatment modality, offers a greater advantage in enhancing the total effective rate. To ensure the robustness of the results, a sensitivity analysis was conducted (see Supplementary Figures). No single study had a significant influence on the results or heterogeneity.

### 3.5 Adverse events

Two studies reported adverse reactions. He et al. (26) reported 5 cases of subcutaneous hemorrhage in the treatment group, while Wang (36) reported 13 cases of mild hemorrhage and 12 cases of needle pain in the treatment group. Adverse reactions in both studies resolved within 1 week, indicating that nape acupuncture may have a favorable safety profile within the scope of the included studies.

### 3.6 Publication bias

For outcome measures with a large number of included studies (*n* ≥ 10), an Egger test was performed to determine whether publication bias was present in the data. Both the total effective rate and the SSA score were tested. Results showed no significant bias for SSA scores (*p* = 0.584 > 0.05) or total effective rate (*p* = 0.052 > 0.05). Funnel plots showed symmetry (see Supplementary Figures), indicating a low likelihood of publication bias.

### 3.7 Trial sequential analysis

**Figure 11:**
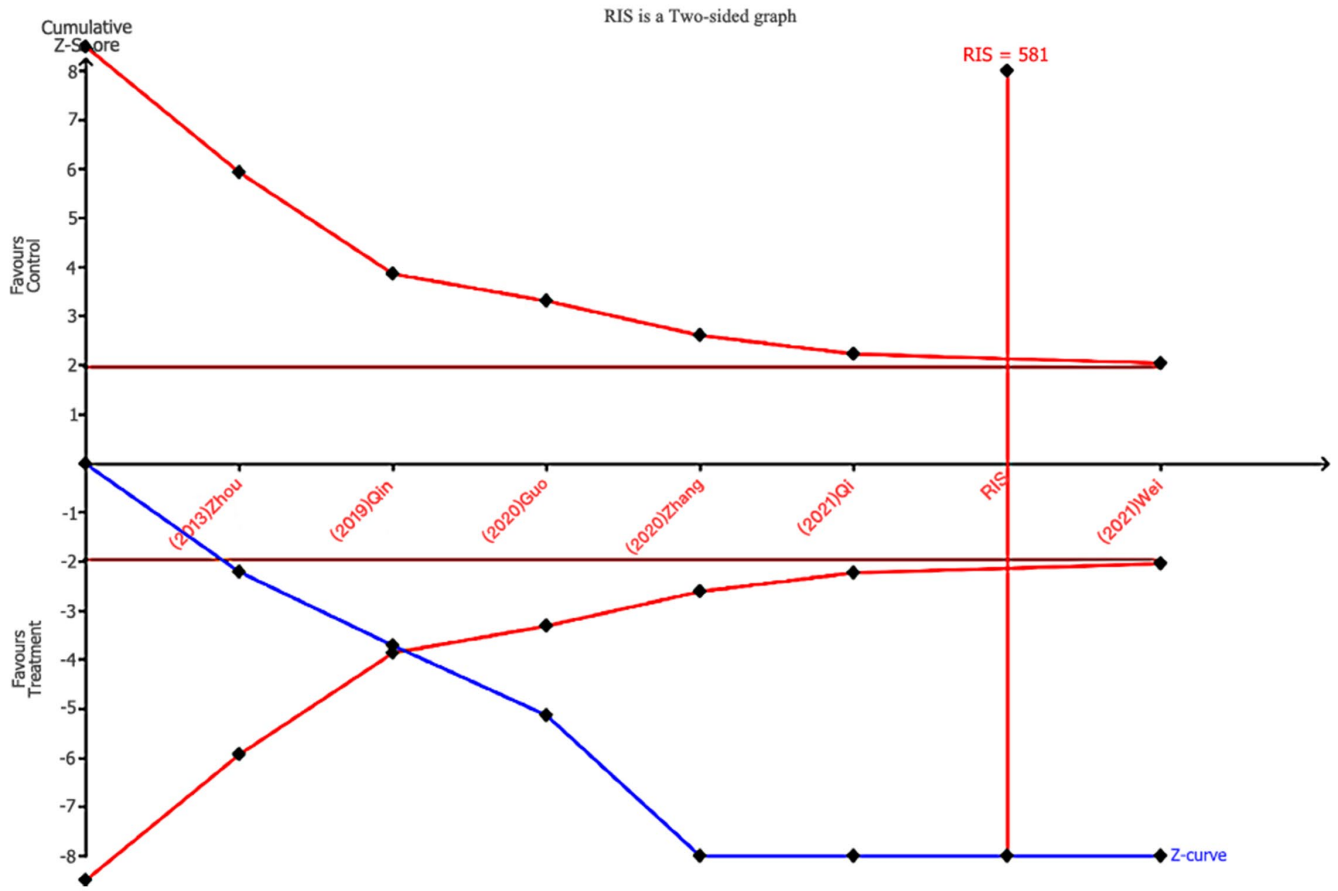
Trial sequential analysis of VFSS.

**Figure 12:**
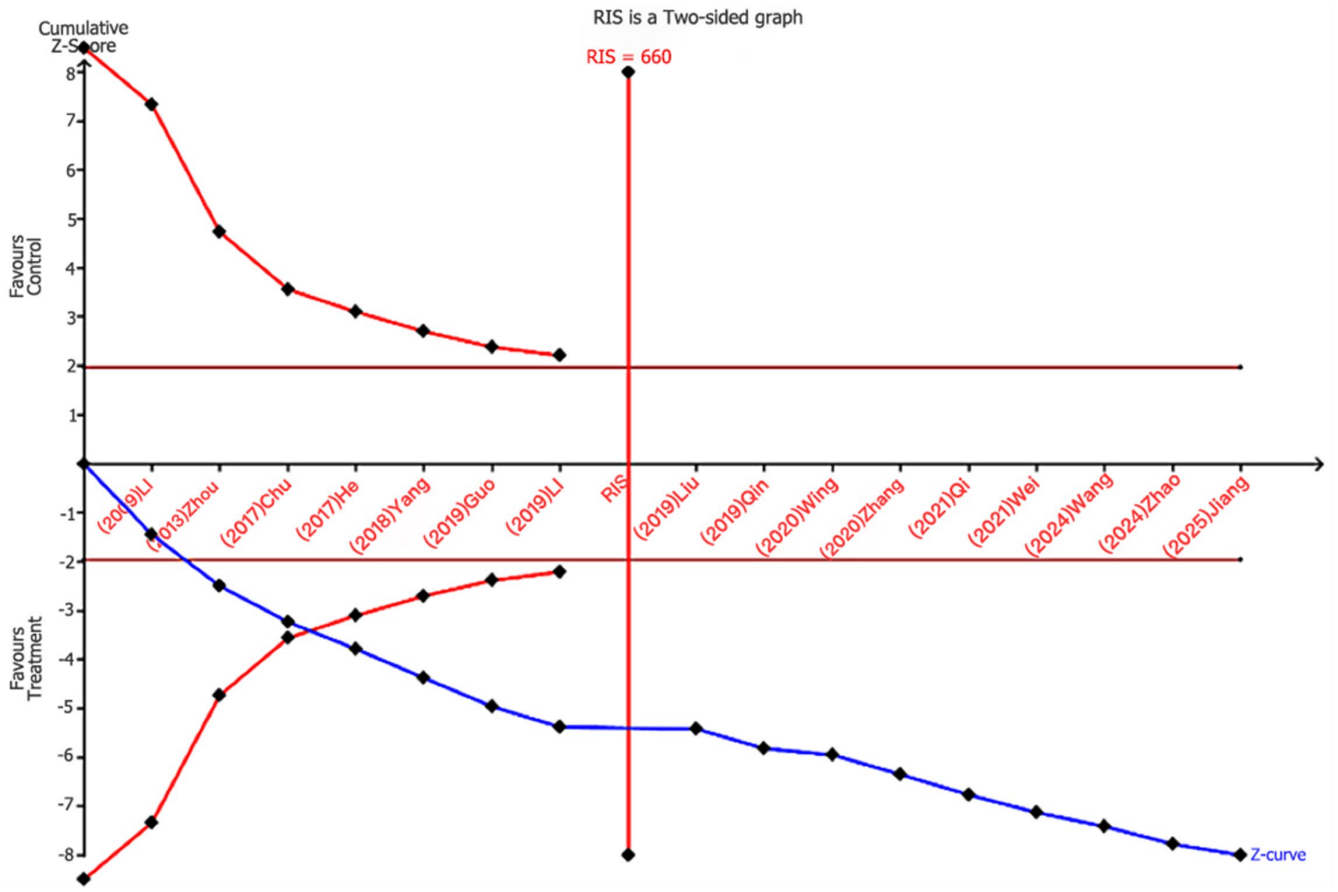
Trial sequential analysis of total effective rates.

TSA analysis was performed for the gold standard measures, VFSS and total effective rate. The TSA results indicated that for VFSS, the cumulative Z-curve crossed the TSMB boundary after the inclusion of the third study and remained stable thereafter. It crossed the ideal number of studies after the inclusion of the sixth trial, confirming that the VFSS metric produced no false positives and that sufficient literature was included, providing ample evidence to support the conclusion (as shown in Figure 11). For the total effective rate indicator, the cumulative Z-curve crossed the TSMB boundary after the fourth study and crossed the ideal number of studies after the eighth study. This indicates that there are no false positives in the total effective rate indicator and provides sufficient evidence to support the conclusion (as shown in Figure 12).

### 3.8 GRADE assessment

Outcome measures were evaluated using the GRADE approach. The evidence level for SSA scores and BI index was rated as very low. The evidence levels for VFSS and SWAL-QOL were both low. The evidence level for the overall clinical response rate was moderate. Results are presented in Table 2.

**Table 2:** Evidence GRADE of included trial outcomes.

Quality assessment							No of patients		Effect			Quality
Outcome	No of studies	Design	Risk of bias	Inconsistency	Indirectness	Imprecision	Other considerations	Treatment	Control	Relative	Absolute	
										(95% CI)		
SSA	15	Randomised trials	Serious^1^	Very serious^2^	No serious indirectness	No serious imprecision	None	724	724	–	MD 3.59 lower (4.35 to 2.84 lower)	⊕OOO
												Very low
VFSS	6	Randomised trials	Serious^1^	Serious^2^	No serious indirectness	No serious imprecision	None	291	290	–	MD 1.22 higher (0.94 to 1.51 higher)	⊕⊕OO
												Low
SWAL-QOL	4	Randomised trials	Serious^1^	Serious^2^	No serious indirectness	No serious imprecision	None	232	233	–	MD 13.42 higher (9.46 to 17.37 higher)	⊕⊕OO
												Low
BI	4	Randomised trials	Serious	Very serious^1^	No serious indirectness	No serious imprecision	None	196	212	–	MD 9.2 higher (3.99 to 14.4 higher)	⊕OOO
												Very low
Total effective rate	16	Randomised trials	Serious^1^	No serious inconsistency	No serious indirectness	No serious imprecision	None	660/724	539/725	OR 3.72 (2.69 to 5.13)	172 more per 1,000 (from 143 more to 194 more)	⊕⊕⊕O
								−91.20%	−74.30%			Moderate
									75%		168 more per 1,000 (from 140 more to 189 more)	

^1^The included studies had incomplete blinding designs.

^2^Literature screening flowchart.

## 4 Discussion

This meta-analysis examined 21 randomized controlled trials (RCTs) involving 1,995 patients with PSD, aiming to systematically evaluate the efficacy of nape acupuncture in improving patients’ quality of life and swallowing function. Results demonstrated that compared to interventions using conventional acupuncture alone, swallowing training, NMES, balloon dilation, or transcranial magnetic stimulation, both nape acupuncture alone and nape acupuncture combined with other therapies significantly improved patients’ swallowing function and quality of life. Notably, PSD patients often experience negative emotions such as anxiety and depression, which can create a vicious cycle and worsen poor outcomes. Using the SWAL-QOL and BI index, this study particularly assessed quality of life improvement as a main outcome measure, while concentrating on clinical indicators such as swallowing function. The SWAL-QOL scale encompasses multiple dimensions, including mental health, feeding time, and fatigue levels. It serves as a specific measurement tool for the quality of life of PSD patients, with its reliability and validity demonstrated in numerous clinical trials, making it a suitable treatment endpoint (44, 45). The BI index is a classic tool for assessing activities of daily living (ADL) in post-stroke patients. It reflects not only self-care abilities but also correlates closely with swallowing recovery, prognosis, and quality of life. To validate the stability of conclusions, sensitivity analyses and Egger’s test were conducted, demonstrating high robustness and no evidence of publication bias. Additionally, the TSA analysis conducted on VFSS scores and total effective rate indicated that the cumulative Z-curves for all outcomes crossed both the conventional threshold and the trial sequential monitoring boundary, suggesting that the existing evidence sufficiently supports a positive effect on PSD. However, due to the study’s lack of specification regarding disease duration and lesion location, as well as potential significant variations in specific needling operations and needle manipulation techniques based on practitioners’ clinical experience, some indicators (e.g., SSA) exhibited high heterogeneity. Consequently, according to the Grade evidence rating system, the evidence level for specific outcome measures was rated as very low.

Modern research indicates that nape acupuncture improves PSD through diverse mechanisms, primarily by promoting neuroplasticity, alleviating ischemia and hypoxia in affected areas, and improving the function of swallowing-related muscle groups. The results of this study indicate that the improvement in VFSS and SSA scores in the nape acupuncture treatment group was significantly greater than that in the control group. This finding can be directly attributed to the activating effect of acupuncture on neural plasticity. Given the unique anatomical locations of commonly used nape acupuncture points, all distributed within sensory fiber territories of the vagus, glossopharyngeal, recurrent laryngeal, and hypoglossal nerves, needling these sites stimulates motor nerve impulse generation. These impulses are transmitted to the cerebral cortex or medullary swallowing center, promoting excitatory signals from the swallowing center and aiding the recovery of damaged neural reflex arc function (46). Combined acupuncture at Fengchi (GB20) and Gongxue (Extra) improves vertebrobasilar artery perfusion, enhances local microcirculation, boosts cellular metabolism, and promotes neuronal axon development. Concurrently, this study employed the SWAL-QOL scale and BI scores to assess quality of life, revealing significant improvements in the treatment group across dimensions, including meal duration, psychological well-being, and activities of daily living. This outcome is closely related to the direct regulatory effects of nape acupuncture on the swallowing muscle groups. For instance, electroacupuncture stimulation at Lianquan (CV23) specifically activates excitatory neurons in layer 5 of the motor cortex (M1). Signals project via the pontine brainstem nucleus (PBN) to the nucleus tractus solitarius (NTS) in the medulla. By regulating activity in swallowing-related muscle groups such as the genioglossus, it promotes recovery of swallowing function (47, 48). At the neurochemical level, nape acupuncture increases the expression of neurotransmitters serotonin (5-HT) and dopamine (DA), thereby enhancing central control over swallowing movements and muscle coordination (49). The excitation-contraction coupling mechanism at the neuromuscular junction, which causes muscle contraction, stimulates the hypoglossal nerve by activating NTS neurons. This releases acetylcholine, improving swallowing function. Coupling mechanism to stimulate neuromuscular junctions and induce muscle contraction, thereby improving swallowing function (50, 51). Furthermore, enhanced swallowing muscle function alleviates anxiety and depression stemming from feeding difficulties, ultimately improving mental health and overall quality of life (52).

This systematic review has several limitations, and its results should be interpreted with caution. Main limitations include: (1) Although a systematic search was performed across multiple Chinese and English databases, all included studies were published in Chinese journals from China, with no rigorously designed studies on the topic identified in English databases. This over-reliance on regionally published journals not indexed internationally introduces language bias and geographical publication bias, substantially limiting the global generalizability of the findings; (2) Owing to limitations in research conditions, grey literature was not retrieved, leading to the potential risk of missing relevant studies; (3) In terms of clinical depth and long-term outcomes, key information was underreported. Most studies provided insufficient details on intervention specifics (e.g., precise acupuncture parameters, individualized acupoint adjustments) and baseline patient characteristics (e.g., neurophysiological subtype of dysphagia, location and type of stroke lesion), hindering in-depth exploration of heterogeneity sources; (4) A majority of the original studies had flaws in methodological design and reporting, which reduced the evidence grade. The procedures for randomization and allocation concealment were poorly described; moreover, most studies failed to implement blinding of outcome assessors, resulting in a significantly elevated risk of performance bias and detection bias. Additionally, the sample sizes were generally small and lacked *a priori* calculation, leading to insufficient statistical power and undermining the credibility of the pooled results; (5) Given the chronic nature of post-stroke dysphagia, the follow-up outcome data of the included studies were inadequate, with a paucity of long-term efficacy data. The study results were thus susceptible to temporal effects, precluding an in-depth evaluation of the persistence of nape acupuncture efficacy.

To mitigate these limitations, we look forward to further research, which will expand searches to include additional databases and languages, thereby encompassing a more racially and culturally diverse population. Establishing standardized guidelines for acupuncture treatments is necessary to reduce treatment variability among practitioners. Implementing more rigorous allocation concealment and blinding designs in clinical trials in order to reduce potential biases.

Such as implementing blinding techniques using sham acupuncture or non-meridian/non-acupoint needling to enhance experimental validity. At the same time, patients’ baseline characteristics should be reported in a standardized and detailed manner, including but not limited to: precise localization and classification of stroke lesions based on neuroimaging, lesion volume, and the severity of dysphagia assessed using standardized tools, such as VFSS, FEES, or the Standardized Swallowing Function Assessment Scale. Establishing follow-up requirements for outcomes to derive long-term efficacy will provide more comprehensive reference information for subsequent research.

## 5 Application to clinical practice

**Figure 13:**
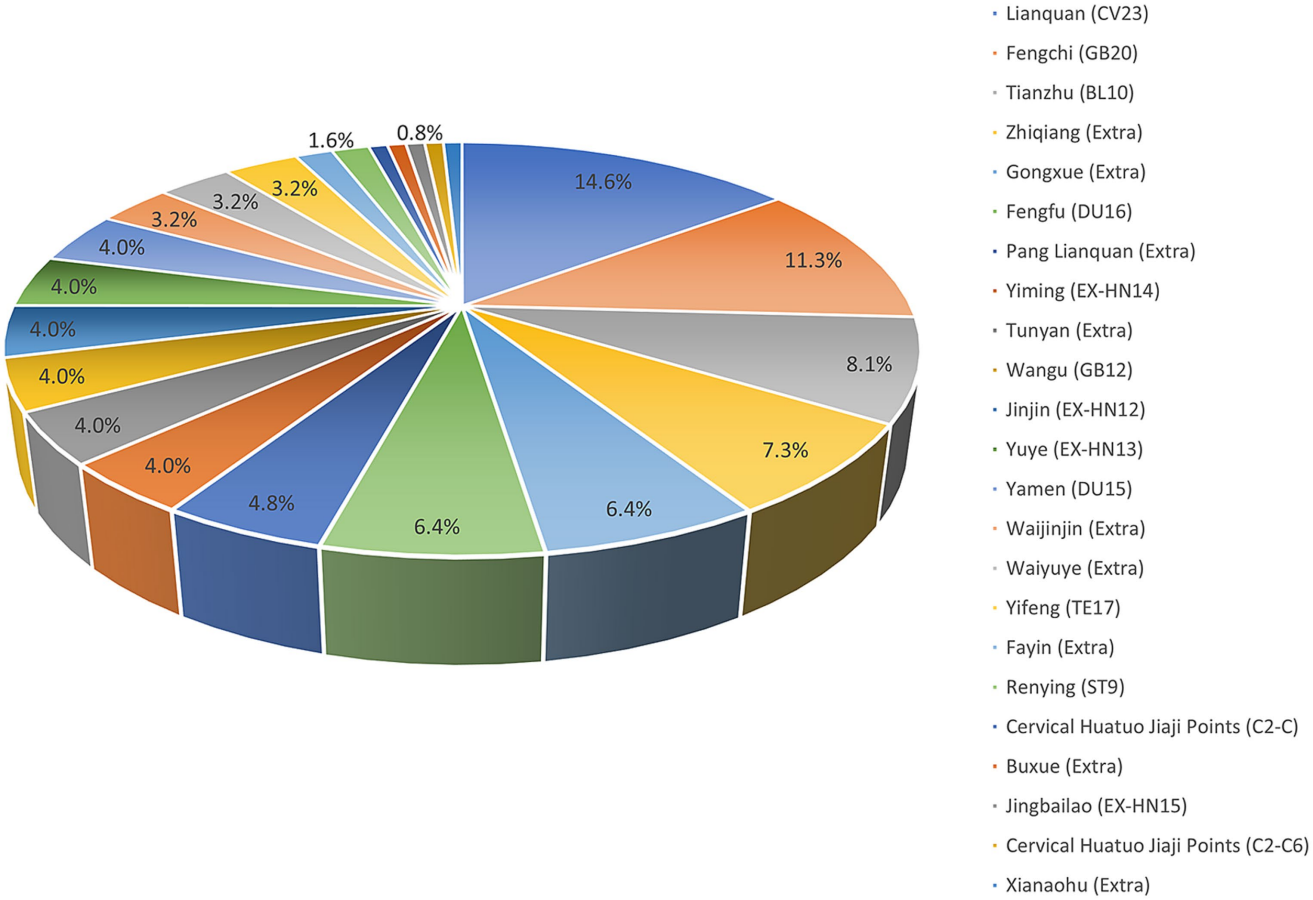
Visualization of acupuncture acupoints.

This study utilized data visualization analysis of acupoint usage in included RCTs (Figure 13), potentially providing reference for developing standardized guidelines on acupuncture treatment for dysphagia following stroke. Statistics indicate that among the 21 included studies, Lianquan (CV23) and Fengchi (GB20) were identified as the most highly concentrated core acupoints. Given that CV23 and GB20 directly target paralyzed or weakened pharyngeal muscles while improving blood supply and neural function in the swallowing center, they can serve as paired acupoints in standardized prescriptions.

Based on these findings, the following indicators are recommended for inclusion in standardized guidelines: 1. Emphasize standardization of acupuncture techniques, including point selection, insertion depth, and manipulation methods. To reduce heterogeneity between studies, future acupuncture research should detail and unify these parameters to ensure valid comparisons across studies. 2. Additionally, treatment protocols for acupuncture should be defined, specifically including key temporal parameters such as treatment frequency (e.g., weekly sessions), duration per session, total number of sessions, and overall treatment duration. Currently, treatment protocols primarily rely on practitioner experience, leading to significant variability across studies. To enhance the reproducibility of clinical research and comparability of results, future studies should explicitly specify and standardize these parameters in protocols. This ensures consistency in intervention dosage across studies, providing a reliable foundation for efficacy comparisons and evidence synthesis. 3. Standardization of control group methodologies is equally essential in acupuncture research design. Placebo acupuncture serves as an effective control group approach, better controlling for placebo effects and providing a foundation for blinding protocols. We recommend adopting this control strategy in future studies to enhance the reliability of results.

## 6 Conclusion

This systematic review, through meta-analysis and TSA results, indicates that the application of the item has a positive effect on improving swallowing function and daily living activities for PSD. Its safety is assured, and the current sample size is sufficient to support the conclusions. However, due to methodological limitations in the included studies, the quality of evidence assessed by GRADE was rated as very low to moderate. This indicates that future research should focus on improving methodology, particularly by implementing blinded designs and incorporating additional follow-up indicators. In the future, multicenter, high-quality randomized controlled trials are warranted to standardize the operational protocols of nape acupuncture for dysphagia post-stroke, to explore its underlying mechanisms in combination with modern technologies, and to investigate its therapeutic efficacy and long-term benefits in special populations. This will enable a more comprehensive evaluation of the treatment’s efficacy for PSD, thereby establishing precise acupuncture prescriptions and treatment guidelines for managing this condition.

## Data Availability

The original contributions presented in the study are included in the article/Supplementary material, further inquiries can be directed to the corresponding author.

## Footnotes

1
http://www.ctu.dk/tsa
