# Supplementary material for: Effectiveness of nape acupuncture for post-stroke dysphagia: a meta-analysis and trial sequential analysis of randomized controlled trials
Source: Front Neurol. 2026 Feb 18;17:1720302. doi: 10.3389/fneur.2026.1720302 (PMC12956699; doi:10.3389/fneur.2026.1720302)

Supplementary Material

Supplementary Figures

Subgroup analysis of SSA: Treatment Duration


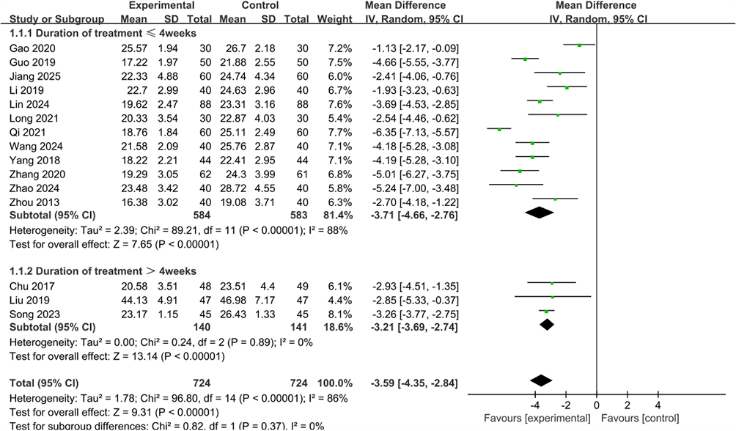


Subgroup analysis of SSA: The control group intervention


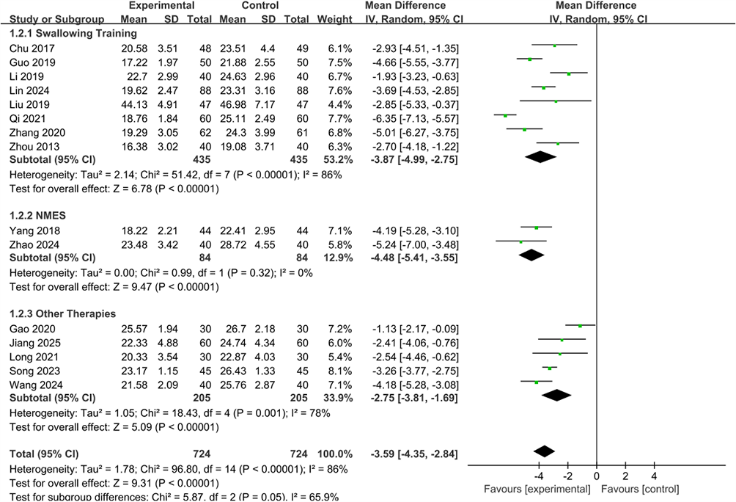


Subgroup analysis of SWAL-QOL: Treatment Duration


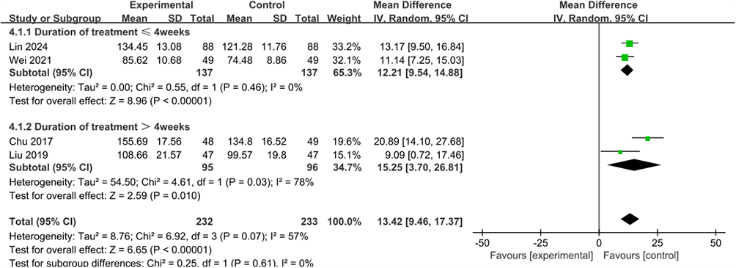


Subgroup analysis of SWAL-QOL: The control group intervention
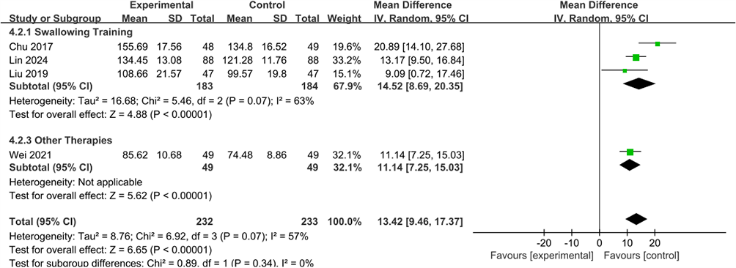


Subgroup analysis of BI: Treatment Duration


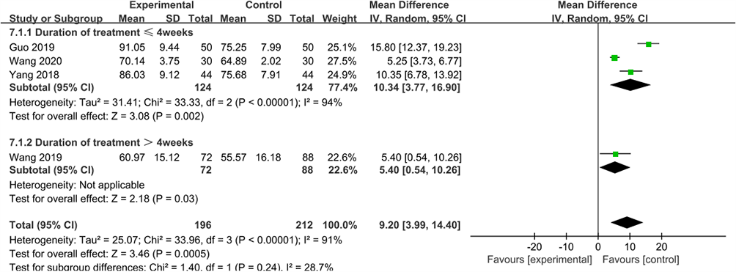


Subgroup analysis of BI: The control group intervention


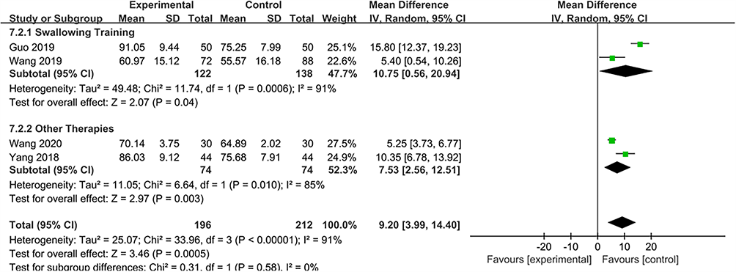


Funnel plots of SSA


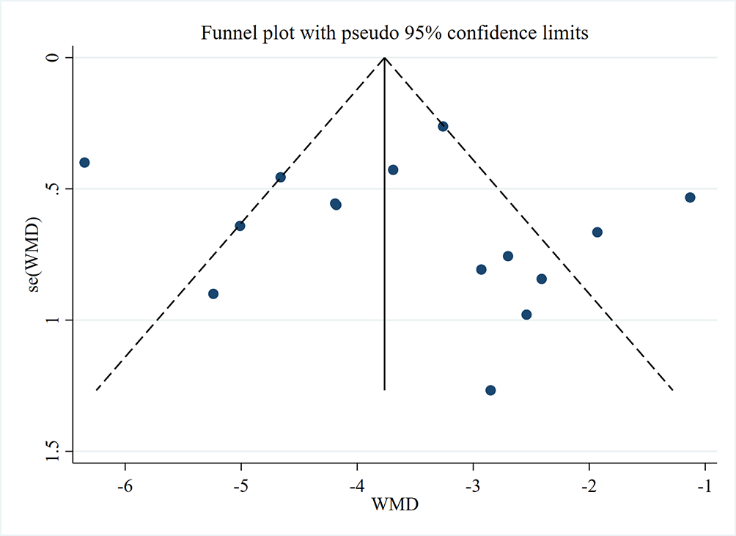


Funnel plots of Total effective rate


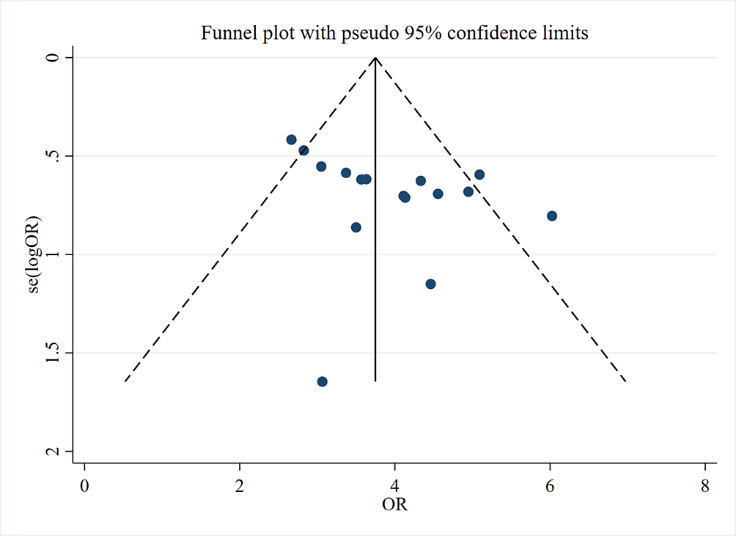


Sensitivity Analysis of VFSS


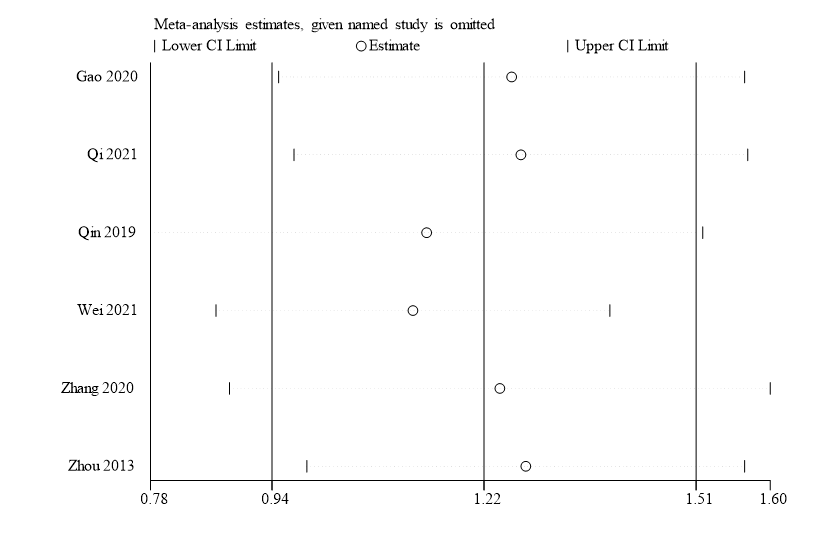


Sensitivity Analysis of SSA
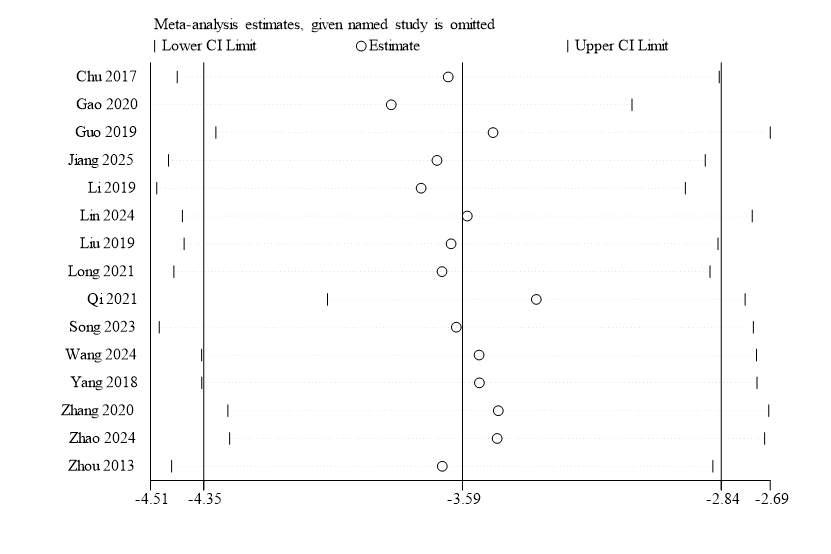


Sensitivity Analysis of SWAL-QOL
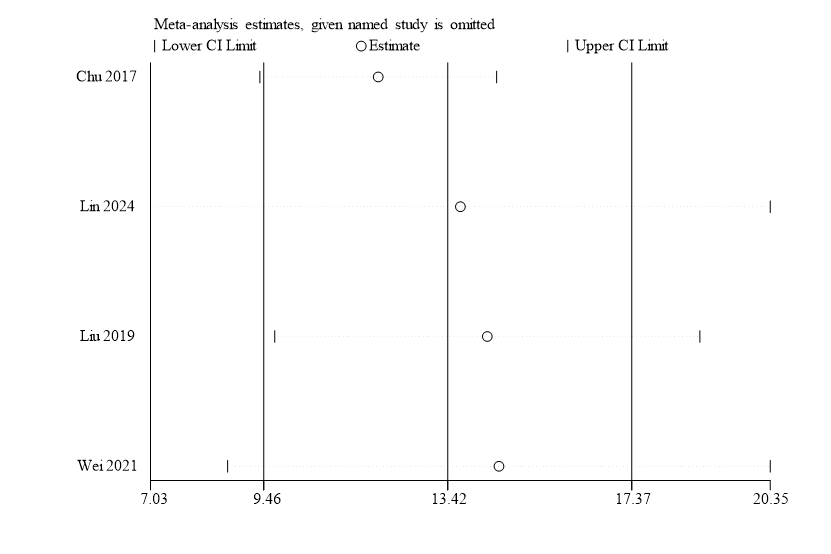


Sensitivity Analysis of BI
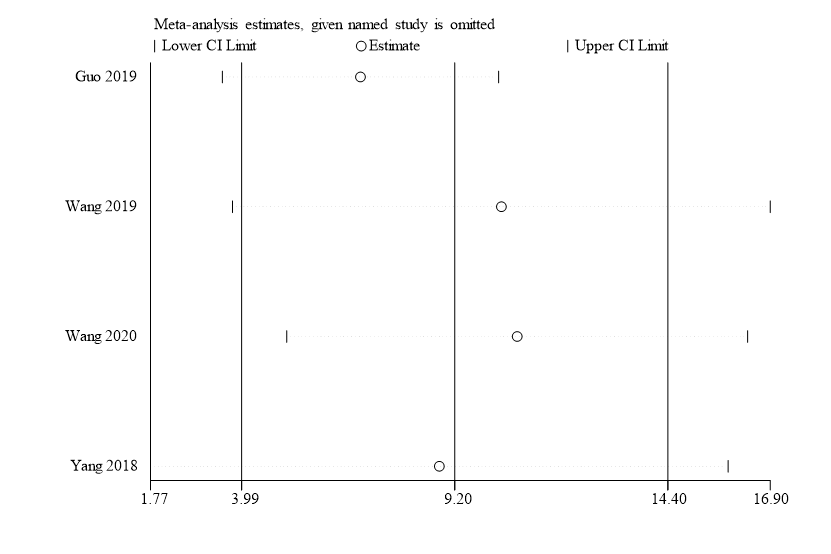


Sensitivity Analysis of Total effective rates


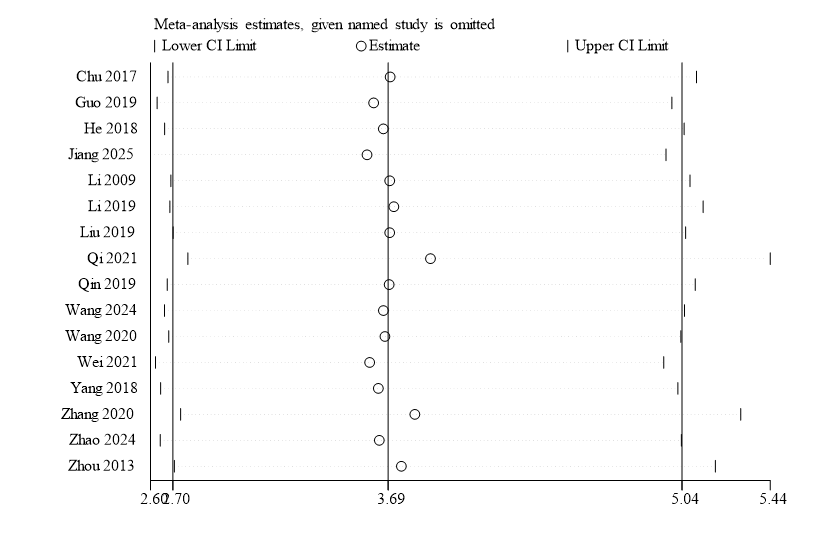

Supplement: Supplementary file 1 [file Supplementary_file_1.docx]
