# Supplementary material for: Effectiveness of nape acupuncture for post-stroke dysphagia: a meta-analysis and trial sequential analysis of randomized controlled trials
Source: Front Neurol. 2026 Feb 18;17:1720302. doi: 10.3389/fneur.2026.1720302 (PMC12956699; doi:10.3389/fneur.2026.1720302)
Supplement: Supplementary file 2 [file Table_1.docx]

Supplementary Material

# Supplementary Tables

English Search Strategy

|  | Search Strategy |
| --- | --- |
| #1 | "Neck Acupuncture" OR "Nape Acupuncture" OR "Neck Needle" OR "Nape Needle" OR "Cervical Acupuncture" |
| #2 | Stroke[Mesh] OR "Stroke" OR "Cerebrovascular Accident" OR "Cerebrovascular Accidents" OR "CVA (Cerebrovascular Accident)" OR "CVAs (Cerebrovascular Accident)" OR "Cerebrovascular Apoplexy" OR "Apoplexy, Cerebrovascular" OR "Vascular Accident, Brain" OR "Brain Vascular Accident" OR "Apoplexy" OR "Cerebral Stroke" OR "Acute Stroke" OR "Acute Strokes" |
| #3 | Deglutition Disorders[Mesh] OR "Deglutition Disorders" OR "Deglutition Disorder" OR "Disorders, Deglutition" OR "Swallowing Disorders" OR "Swallowing Disorder" OR "Dysphagia" OR "Oropharyngeal Dysphagia" OR "Dysphagia, Oropharyngeal" OR "Esophageal Dysphagia" OR "Dysphagia, Esophageal" |
| #4 | Randomized Controlled Trial [Publication Type] |
| #5 | "Randomized Controlled Trial" OR "randomized" OR "placebo" OR "RCT" |
| #6 | #4 OR #5 |
| #7 | #1 AND #2 AND #3 AND #6 |

Chinese Search Strategy

|  | Search Strategy |
| --- | --- |
| #1 | 题名或关键词 = 颈针 OR 题名或关键词 = 颈部针 OR 题名或关键词 = 颈项针 OR 题名或关键词 = 项针 OR 题名或关键词 = 颈部针法 OR 题名或关键词 = 颈部针刺 OR 题名或关键词 = 颈针疗法 [题名或关键词] |
| #2 | 题名或关键词 = 卒中 OR 题名或关键词 = 脑中风 OR 题名或关键词 = 急性卒中 OR 题名或关键词 = 脑卒中 OR 题名或关键词 = 脑血管意外 OR 题名或关键词 = 脑血管中风 OR 题名或关键词 = CVA OR 题名或关键词 = CVAs OR 题名或关键词 = 中风，急性 OR 题名或关键词 = 出血性脑卒中 OR 题名或关键词 = 缺血性脑卒中 [题名或关键词] |
| #3 | 题名或关键词 = 吞咽障碍 OR 题名或关键词 = 咽下障碍 OR 题名或关键词 = 吞咽困难 OR 题名或关键词 = 口咽性咽下困难 OR 题名或关键词 = 食管咽下困难 [题名或关键词] |
| #4 | 题名或关键词 = 随机对照试验 OR 题名或关键词 = 随机对照实验 OR 题名或关键词 = 随机对照研究 OR 题名或关键词 = 随机对照 OR 题名或关键词 = RCT OR 题名或关键词 = 临床研究 OR 题名或关键词 = 临床试验 OR 题名或关键词 = 临床观察 [题名或关键词] |
| #5 | #1 AND #2 AND #3 AND #4 |
